# Phase Ib study of avadomide (CC‐122) in combination with rituximab in patients with relapsed/refractory diffuse large B‐cell lymphoma and follicular lymphoma

**DOI:** 10.1002/jha2.394

**Published:** 2022-02-14

**Authors:** Loretta J. Nastoupil, John Kuruvilla, Julio C. Chavez, Fontanet Bijou, Thomas E. Witzig, Armando Santoro, Ian W. Flinn, Carola Boccomini, Vaishalee P. Kenkre, Paolo Corradini, Iris Isufi, David J. Andorsky, Leonard M. Klein, Daniel R. Greenwald, Randeep Sangha, Frank Shen, Patrick Hagner, Yan Li, Juergen Dobmeyer, Nian Gong, Shailaja Uttamsingh, Michael Pourdehnad, Vincent Ribrag

**Affiliations:** ^1^ Department of Lymphoma and Myeloma Division of Cancer Medicine MD Anderson Cancer Center The University of Texas Houston Texas USA; ^2^ Division of Medical Oncology and Hematology Princess Margaret Cancer Centre University of Toronto Toronto Ontario Canada; ^3^ H. Lee Moffitt Cancer Center and Research Institute Tampa Florida USA; ^4^ Institut Bergonié Bordeaux Cedex France; ^5^ Mayo Clinic Rochester Minnesota USA; ^6^ Department of Biomedical Sciences Pieve Emanuele Milan Humanitas University Italy ‐IRCCS Humanitas Research Hospital‐ Humanitas Cancer Center Rozzano Milan Italy; ^7^ Sarah Cannon Research Institute Nashville Tennessee USA; ^8^ SC Ematologia ASOU Città della Salute e della Scienza di Torino Turin Italy; ^9^ Division of Hematology and Oncology University of Wisconsin Madison Wisconsin USA; ^10^ IRCCS Istituto Nazionale dei Tumori University of Milano Milano Italy; ^11^ Yale Cancer Center New Haven Connecticut USA; ^12^ Rocky Mountain Cancer Centers The US Oncology Network Boulder Colorado USA; ^13^ Illinois Cancer Specialists The US Oncology Network Niles Illinois USA; ^14^ Cancer Center of Santa Barbara Santa Barbara California USA; ^15^ Cross Cancer Institute Edmonton Alberta Canada; ^16^ Bristol Myers Squibb Princeton New Jersey USA; ^17^ Centre for Innovation and Translational Research Europe (CITRE) Bristol‐Myers Squibb Company Seville Spain; ^18^ Institut Gustave Roussy Villejuif France

**Keywords:** avadomide, CELMoD, diffuse large B‐cell lymphoma, follicular lymphoma

## Abstract

The multicenter, phase Ib CC‐122‐DLBCL‐001 dose‐expansion study (NCT02031419) explored the cereblon E3 ligase modulator (CELMoD) agent avadomide (CC‐122) plus rituximab in patients with relapsed/refractory (R/R) diffuse large B‐cell lymphoma (DLBCL) or follicular lymphoma (FL). Patients received avadomide 3 mg/day 5 days on/2 days off plus rituximab 375 mg/m^2^ on day 8 of cycle 1, day 1 of cycles 2 through 6, and day 1 of every third subsequent cycle for 2 years. Primary endpoints were safety and tolerability; preliminary efficacy was a secondary endpoint. A total of 68 patients were enrolled (DLBCL [*n* = 27], FL [*n* = 41; 31 lenalidomide‐naïve, 10 lenalidomide‐treated]). Median age was 62 years (range, 33–84 years), and patients had received a median of 3 (range, 1–8) prior regimens. Among patients with DLBCL, 66.7% had primary refractory disease (partial response or less to initial therapy). Among patients with FL, 65.9% were rituximab‐refractory at study entry and 10.0% were lenalidomide‐refractory. The most common any‐grade avadomide‐related adverse events (AEs) were neutropenia (63.2%), infections/infestations (23.5%), fatigue (22.1%), and diarrhea (19.1%). The most common grade 3/4 avadomide‐related AEs were neutropenia (55.9%) infections/infestations (8.8%), and febrile neutropenia (7.4%). In patients with DLBCL, overall response rate (ORR) was 40.7% and median duration of response (mDOR) was 8.0 months. In patients with FL, ORR was 80.5% and mDOR was 27.6 months; response rates were similar in lenalidomide‐naïve and ‐treated patients. Avadomide plus rituximab was well tolerated, and preliminary antitumor activity was observed in patients with R/R DLBCL and FL, including subgroups with typically poor outcomes. These results support further investigation of novel CELMoD agents in combination with rituximab in R/R DLBCL and FL.

## 1 INTRODUCTION

Non‐Hodgkin lymphoma (NHL) includes a diverse spectrum of lymphoproliferative diseases, with approximately 85%–90% originating from B cells [1]. Diffuse large B‐cell lymphoma (DLBCL) and follicular lymphoma (FL) are the most common forms of B‐cell NHL [1, 2, 3, 4, 5]. DLBCL is characterized by aggressive clinical behavior [2] and comprises two major molecular subtypes (germinal center B‐cell–like [GCB] and activated B‐cell–like [ABC]) that are associated with differential response to treatment [6]. FL is an indolent form of NHL, and although it generally responds well to treatment, the disease course is characterized by repeated relapses interspersed with treatment‐free intervals of progressively shorter duration [4].

Treatment options such as immunochemotherapy with rituximab, cyclophosphamide, doxorubicin, vincristine, and prednisone (R‐CHOP) are standard therapy in early treatment lines and have improved the prognosis of patients with NHL [7, 8]. However, various factors have been associated with poor prognosis. Patients with ABC DLBCL have less favorable outcomes than those with GCB DLBCL when treated with standard immunochemotherapy [9]. In addition, patients with primary refractory disease (defined as relapse < 12 months after diagnosis) or “double‐hit” lymphomas (characterized by gene rearrangements of *MYC* and B‐cell lymphoma 2 [*BCL2*] or *BCL6*) have poor outcomes despite salvage treatment and autologous stem cell transplant (ASCT) [10, 11]. Patients with FL whose disease progresses within 2 years of initial diagnosis or whose disease is double‐refractory to both rituximab and chemotherapy also typically have a poor prognosis [12]. The poor response to immunochemotherapy seen in patients with relapsed/refractory (R/R) disease has led to considerable interest in treatment approaches based on targeted therapy combinations [13, 14].

Avadomide (CC‐122) is a novel cereblon E3 ligase modulator (CELMoD) agent that binds to cereblon in the cullin 4 E3 ubiquitin ligase complex and promotes ubiquitination and degradation of the hematopoietic transcription factors Aiolos and Ikaros [15]. This leads to de‐repression of interferon‐response gene promoters, apoptosis of malignant B cells, and de‐repression of cytokine promoters such as IL‐2, resulting in T‐cell activation [15, 16, 17]. Preclinical studies have shown avadomide to be a more potent CELMoD agent than lenalidomide, demonstrating greater induction of Aiolos and Ikaros degradation and greater increases in tumor cell apoptosis in vitro, and avadomide, but not lenalidomide, has been shown to increase the relative abundance of interferon‐stimulated proteins in DLBCL models [15]. Avadomide has also shown antiproliferative activity in both ABC and GCB DLBCL cell lines, while lenalidomide has preferential activity in ABC DLBCL cell lines [15, 18]. Avadomide has shown promising preclinical activity and phase I clinical efficacy as a monotherapy in NHL and combination with the anti‐CD20 monoclonal antibody obinutuzumab in R/R DLBCL and FL [19, 20]. Furthermore, preclinical studies have demonstrated that avadomide in combination with rituximab is synergistic in a lymphoma model [21]. These data support the evaluation of rituximab in combination with avadomide in R/R DLBCL and FL.

The dose‐escalation portion of the CC‐122‐DLBCL‐001 study, published as a companion article in this issue of *eJHaem*, evaluated combinations of avadomide, the mammalian target of rapamycin kinase inhibitor CC‐223, and the Bruton's tyrosine kinase (BTK) inhibitor CC‐292, administered as doublets and as triplets in combination with rituximab in patients with R/R DLBCL [21]. The combination of avadomide and rituximab was selected for dose‐expansion based on its preliminary antitumor activity and safety profile in patients with R/R DLBCL. Here, we report results from the dose‐expansion portion (part B) of the study, which evaluated this combination in patients with R/R DLBCL and FL.

## 2 MATERIALS AND METHODS

### 2.1 Study design

CC‐122‐DLBCL‐001 (NCT02031419, 2013‐001501‐81) is a phase Ib, multicenter, open‐label study of avadomide, CC‐223, CC‐292, and rituximab in R/R DLBCL and FL. The study consists of two parts: a dose‐escalation phase that included all study drugs [21] and a dose‐expansion phase that examined avadomide in combination with rituximab. Here we report results from the dose‐expansion phase, where patients received avadomide (3 mg avadomide formulated capsule dosed daily for five consecutive days out of 7 days [5/7 days] per week) in combination with rituximab 375 mg/m^2^ administered once per 28‐day cycle (day 8 of cycle 1, day 1 of cycles 2–6, and day 1 of every third subsequent cycle for 2 years; Figure S1). The avadomide dose was selected based on safety, pharmacokinetic, and pharmacodynamic data from previous avadomide clinical studies [19, 22]. The study was conducted in accordance with the principles of the Declaration of Helsinki and in adherence to the E6 guideline for Good Clinical Practice delineated by the International Council for Harmonisation. The protocol was reviewed and approved by each site's Institutional Review Board or Independent Ethics Committee prior to initiation of the study, and all patients provided written informed consent.

The primary objectives were to determine the safety and tolerability of avadomide when administered in combination with rituximab. Secondary objectives were to determine the preliminary efficacy of the drug combination.

### 2.2 Patients

Eligible patients were aged 18 years or older with histologically or cytologically confirmed R/R DLBCL (including transformed indolent lymphoma) or R/R CD20‐positive FL. All DLBCL patients were chemorefractory, defined as having stable disease (SD) ≤ 12 months or progressive disease (PD) as the best response to their last chemotherapy and/or PD or recurrence within 12 months of prior ASCT. Patients must have received an anti‐CD20 monoclonal antibody therapy (unless tumor was CD20‐negative) and anthracycline‐containing chemotherapy, as well as ≥1 prior salvage treatment (unless ineligible for ASCT). Both lenalidomide‐naïve and ‐exposed patients were included in the FL population. Lenalidomide‐naïve FL (FL‐1) patients received at least one prior standard systemic treatment regimen, including systemic chemo‐, immune‐, or chemo‐immunotherapy, and at least one prior line of salvage therapy or were double‐refractory, with no prior exposure to lenalidomide. Lenalidomide‐exposed FL (FL‐2) patients were previously treated with at least two cycles of lenalidomide‐containing regimen, either as monotherapy or in combination, and experienced relapse within 1 year of the last dose of lenalidomide following initial response of complete response (CR) to lenalidomide, progression within 1 year of the last dose of lenalidomide following initial response of PR to lenalidomide, or were disease‐refractory to lenalidomide treatment. All patients had measurable disease of >1.5 cm (long axis) or >1.0 cm (both long and short axes). Patients with symptomatic central nervous system disease were excluded.

Patients were considered to be rituximab‐refractory if they did not have a CR or PR during, or had PD within 6 months of completing, treatment comprising ≥ 4 doses of rituximab monotherapy or ≥ 2 doses of rituximab in combination with chemotherapy ≥ 375 mg/m^2^ administered weekly, or had PD during or within 6 months of completing rituximab maintenance therapy. Patients refractory to both rituximab and an alkylating agent were considered to be double‐refractory, with refractoriness to alkylating agents defined as lack of a CR or PR during, or PD within 6 months of completing, a regimen of alkylating agent‐containing chemotherapy comprising ≥ 2 cycles of treatment.

### 2.3 Treatment

In the dose‐expansion part of this study, all patients received 3 mg of oral avadomide formulated capsule (recommended phase 2 dose [RP2D]) given intermittently for five consecutive days of 7 days per week (5/7 days) in combination with 375 mg/m^2^ rituximab, administered intravenously, once per 28‐day cycle for the first six cycles and once every third subsequent cycle for 2 years. The intermittent avadomide dosing schedule was shown to improve tolerability and reduced the frequency and severity of neutropenia associated with avadomide monotherapy [20]. Dose reductions were permitted for avadomide at any cycle; no dose reductions were allowed for rituximab, but treatment could be discontinued at the discretion of the investigator. Any study drug–related toxicity meeting the dose‐limiting toxicity (DLT) criteria required a dose reduction or interruption. DLTs are defined in the Supporting Information. Routine prophylactic use of growth factors for hematological treatment‐emergent adverse events (TEAEs; i.e., granulocyte colony‐stimulating factor [G‐CSF] and granulocyte‐macrophage CSF [GM‐CSF]) was permitted from cycle 2 onward. Study treatment was given until disease progression, unacceptable toxicity, or patient/physician decision to withdraw consent.

### 2.4 Study assessments

AEs were assessed according to the National Cancer Institute Common Terminology Criteria for AEs, version 4.03, at least 28 days after the last dose. Blood samples for pharmacokinetic analysis were collected <15 min prior to dosing (pre‐dose) and at 1.5 and 3‐h post‐dose on days 1 and 15 of cycle 1. Responses were assessed by the investigator per International Working Group 2007 criteria [23]. Tumor assessments, including computed tomography (CT) of the chest, abdomen, and pelvis, and fluorodeoxyglucose positron emission tomography were performed at screening, with a brain scan (CT or magnetic resonance imaging) for patients with known cerebral involvement. Baseline tumor biomarkers to determine cell of origin (COO) and tumor microenvironment gene classifier in DLBCL were assessed via RNA expression profiling using NanoString technology. The development of the gene classifier was previously described by Risueno et al [24].

### 2.5 Statistical analyses

Safety analyses were performed on all patients who received ≥1 dose of study treatment. Efficacy evaluable patients were those who completed ≥1 treatment cycle and had a baseline and ≥1 postbaseline efficacy assessment. The pharmacokinetic‐evaluable population comprised all patients who took ≥1 dose of study treatment and had evaluable concentration data to determine the pharmacokinetic parameters. Safety and efficacy analyses were based on the safety population except where noted (pharmacokinetics). The median duration of response (mDOR), median progression‐free survival (PFS), and median overall survival (OS) were calculated per the Kaplan–Meier method. All statistical tests were conducted with a two‐sided significance level of 0.05. No adjustment was made for multiple comparisons/multiplicity.

## 3 RESULTS

### 3.1 Patients

From August 10, 2016, to August 27, 2018, 68 patients, 27 with DLBCL and 41 with FL, were enrolled in the dose‐expansion phase. Of the 41 patients with FL, 31 were lenalidomide‐naïve and 10 were lenalidomide‐exposed. As of January 10, 2020, cutoff date, 54 patients (79.4%) had discontinued study treatment and 14 (20.6%) were ongoing (Table S1). The most common reason for treatment discontinuation was PD (*n *= 29 [42.6%]), followed by AE (*n *= 16 [23.5%]), physician decision (*n *= 6 [8.8%]), death (*n *= 1 [1.5%]), and other (*n *= 2 [2.9%]) (Table S1). Patient demographics and baseline characteristics are shown in Table 1. The median age was 62 years (range, 33–84 years), and 63.2% were male. Twenty‐five patients (36.8%) had an Eastern Cooperative Oncology Group performance status (ECOG PS) score of 0, and 43 patients (63.2%) had an ECOG PS score of 1. At enrollment, 47 patients (69.1%) had stage III/IV disease. Patients had received a median of 3 (range, 1–8) prior systemic anticancer therapies, and seven (10.3%) had received prior ASCT. None of the patients had received chimeric antigen receptor (CAR) T‐cell therapies. Of the 27 DLBCL patients, eight (29.6%) had transformed DLBCL, 19 (70.4%) had de novo DLBCL, 18 (66.7%) had primary refractory DLBCL, and two (7.4%) had double‐hit disease. COO classification, determined by NanoString technology, identified 11 patients (40.7%) with the GCB subtype, three patients (11.1%) with the ABC subtype, and 13 patients (48.1%) as unclassified/missing. Twenty‐one patients (77.8%) had high‐ or high‐intermediate risk disease, with an International Prognostic Index (IPI) score ≥3. Of the 41 FL patients, 13 (31.7%) had double‐refractory disease, three (7%) had bulky disease (defined as tumor size of ≥7 cm), and four (10%) had lenalidomide‐refractory disease. Ten patients (24.4%) had a high‐risk Follicular Lymphoma IPI‐1 (FLIPI‐1) score, and nine (22.0%) had an intermediate‐risk FLIPI‐1 score. Per protocol, all patients in both DLBCL and FL cohorts were exposed to rituximab.

**TABLE 1:** Patient baseline characteristics

**Characteristic**		**DLBCL (*N* = 27)**	**FL Overall (*N *= 41)**	**Overall (*N* = 68)**
Age in years, median (range)		63 (33–84)	61 (41–81)	62 (33‐84)
Age > 65 years, *n* (%)		12 (44.4)	10 (24.4)	22 (32.4)
Male, *n* (%)		20 (74.1)	23 (56.1)	43 (63.2)
ECOG PS, *n* (%)	0	5 (18.5)	20 (48.8)	25 (36.8)
	1	22 (81.5)	21 (51.2)	43 (63.2)
No. of prior systemic anticancer regimens, median (range)		3 (1–6)	3 (1–8)	3 (1–8)
Disease stage III/IV,^a^ *n* (%)		22 (81.5)	25 (61.0)	47 (69.1)
Prior ASCT, *n* (%)		3 (11.1)	4 (9.8)	7 (10.3)
De novo DLBCL, *n* (%)		19 (70.4)	–	–
Transformed DLBCL, *n* (%)		8 (29.6)	–	–
DLBCL cell of origin,^b^ *n* (%)	GCB	11 (40.7)	–	–
	ABC	3 (11.1)	–	–
	Unclassified	3 (11.1)	–	–
DLBCL tumor microenvironment gene classifier group,^b^ *n* (%)	Positive	7 (25.9)	–	–
	Negative	10 (37.0)	–	–
Double‐hit lymphoma,^c^ *n* (%)		2 (7.4)	–	–
Primary refractory DLBCL, *n* (%)		18 (66.7)	–	–
IPI, *n* (%)	Low (0–1)	2 (7.4)		
	Low intermediate (2)	4 (14.8)		
	High intermediate (3)	11 (40.7)		
	High (4‐5)	10 (37.0)		
FL history, *n* (%)	Bulky disease^d^	–	3 (7.3)	–
	Rituximab refractory	–	27 (65.9)	–
	Refractory to an alkylating agent	–	13 (31.7)	–
	Double‐refractory^e^	–	13 (31.7)	–
	Lenalidomide‐refractory^f^	–	4 (10.0)	
FLIPI‐1, *n* (%)	Low risk (0–1)	–	2 (4.9)	–
	Intermediate risk (2)	–	9 (22.0)	–
	High risk (≥3)	–	10 (24.4)	–
	Not done	–	8 (19.5)	–
	Unknown	–	12 (29.3)	–

Data cutoff: January 10, 2020.

^a^ Disease stage in FL was classified using the Ann Arbor staging system.

^b^ Data missing for 10 patients.

^c^ Double‐hit lymphomas have *MYC* and either *BCL2* or *BCL6* rearrangements or overexpression.

^d^ Bulky disease defined as a tumor size of ≥7 cm.

^e^ Double‐refractory was defined as refractory to both rituximab and an alkylating agent.

^f^ Lenalidomide‐refractory was defined as the best response of stable disease or progression to lenalidomide therapy.

Abbreviations: ABC, activated B‐cell; ASCT, autologous stem cell transplantation; DLBCL, diffuse large B‐cell lymphoma; ECOG PS, Eastern Cooperative Oncology Group performance status; FL, follicular lymphoma; GCB, germinal center B‐cell; IPI, International Prognostic Index.

### 3.2 Treatment

As shown in Table S2, the overall median duration of avadomide treatment was 274.5 days (range, 7–1199 days) and the median relative dose intensity was 0.99 (range, 0.5–1.0). The overall median duration of rituximab treatment was 245 days (range, 21–609 days). The median number of treatment cycles was 9.0 (range, 0–42) for avadomide and 9.0 (range, 1–22) for rituximab.

### 3.3 Safety

TEAEs, listed in Table S3, mainly consisted of hematologic and gastrointestinal events. Overall, the most common any‐grade and grade 3/4 TEAE was neutropenia (*n* = 44, 64.7%; *n *= 39, 57.4%). TEAEs were mitigated by dose modifications, and neutropenia was effectively managed by treatment with growth factors, with concomitant growth factor use reported in 33 patients (48.5%).

Any‐grade treatment‐related AEs (TRAEs) occurring in ≥10% of patients and grade 3/4 TRAEs occurring in at least 2 patients are reported in Table 2. Overall, 63 patients (92.6%) had at least one avadomide‐related AE, and 46 (66.2%) patients had at least one grade 3/4 avadomide‐related AE. The most common any‐grade and grade 3/4 avadomide‐related AEs were neutropenia (any‐grade: *n* = 43, 63.2%; grade 3/4: *n *= 38, 55.9%) and infections/infestations (any‐grade: *n *= 16, 23.5%; grade 3/4: *n *= 6, 8.8%). Febrile neutropenia was a grade 3/4 TRAE in five patients (7.4%), all in the FL cohort.

**TABLE 2:** Treatment‐related adverse events

	Related to any study drug (*N =* 68)		Avadomide‐related (*N =* 68)		Rituximab‐related (*N =* 68)	
TRAE, *n* (%)	Any grade	Grade 3/4	Any grade	Grade 3/4	Any grade	Grade 3/4
**≥1 TRAE**	64 (94.1)	46 (67.6)	63 (92.6)	45 (66.2)	45 (66.2)	30 (44.1)
Neutropenia	43 (63.2)	38 (55.9)	43 (63.2)	38 (55.9)	22 (32.4)	20 (29.4)
Infections and infestations^a^	20 (29.4)	8 (11.8)	16 (23.5)	6 (8.8)	16 (23.5)	6 (8.8)
Fatigue	15 (22.1)	2 (2.9)	15 (22.1)	2 (2.9)	8 (11.8)	2 (2.9)
Diarrhea	13 (19.1)	2 (2.9)	13 (19.1)	2 (2.9)	6 (8.8)	1 (1.5)
Rash	11 (16.2)	1 (1.5)	9 (13.2)	1 (1.5)	4 (5.9)	0
Nausea	11 (16.2)	0	11 (16.2)	0	4 (5.9)	0
Rash maculopapular	9 (13.2)	0	8 (11.8)	0	1 (1.5)	0
Infusion‐related reactions	8 (11.8)	1 (1.5)	NA	NA	8 (11.8)	1 (1.5)
Cough	8 (11.8)	0	6 (8.8)	0	6 (8.8)	0
Constipation	7 (10.3)	0	7 (10.3)	0	1 (1.5)	0
Asthenia	7 (10.3)	0	6 (8.8)	0	2 (2.9)	0
Muscle spasms	7 (10.3)	0	7 (10.3)	0	5 (7.4)	0
Febrile neutropenia	5 (7.4)	5 (7.4)	5 (7.4)	5 (7.4)	2 (2.9)	2 (2.9)
Lipase increased	5 (7.4)	4 (5.9)	5 (7.4)	4 (5.9)	3 (4.4)	2 (2.9)
Leukopenia	4 (5.9)	3 (4.4)	4 (5.9)	3 (4.4)	1 (1.5)	0
Lymphopenia	3 (4.4)	3 (4.4)	3 (4.4)	3 (4.4)	2 (2.9)	2 (2.9)

Data cutoff: January 10, 2020.

Any‐grade TRAEs reported in ≥10% of patients or at grade 3/4 severity in >2 patients in the overall dose expansion population (DLBCL and FL).

^a^ Infections and infections, System Organ Class including upper respiratory tract infection, lung infection, pneumonia, bronchitis, urinary tract infection, progressive multifocal leukoencephalopathy, abscess, body tinea, cellulitis, diverticulitis, herpes simplex, oral candidiasis, rash pustular, rectal abscess, respiratory tract infection, and sepsis.

Abbreviations: DLBCL, diffuse large B‐cell lymphoma; FL, follicular lymphoma; NA, not applicable; TRAE, treatment‐related adverse event.

Serious AEs (SAEs) are reported in Table S4. Overall, 32 patients (47.1%) experienced serious TEAEs. The most common any‐grade SAE was febrile neutropenia (*n *= 5, 7.4%) followed by pneumonia (*n *= 4, 5.9%). SAEs related to avadomide were reported in 11 patients (16.2%), including one patient with DLBCL (3.7%) and 10 patients with FL (24.4%). The most common SAEs (≥1 patient) related to avadomide were febrile neutropenia in 3 patients and pneumonia in two patients.

Of the 68 patients overall, 61 (89.7%) had at least one dose interruption of avadomide and 42 (61.8%) had at least one dose interruption of rituximab; both were mostly due to AEs (48 of 61 patients with avadomide interruptions [78.7%] and 31 of 42 patients with rituximab interruptions [73.8%], respectively). Fifteen (22.1%) patients had at least one dose reduction of avadomide, 14 of 15 (93.3%) of which were due to AEs (Table S2). Rituximab dose reductions were not allowed in this study. As of January 10, 2020, cutoff date, 54 patients discontinued treatment, 16 of whom discontinued due to AEs (Table S1).

Overall, 22 deaths were reported, 21 of which occurred during follow‐up. Four patients had TEAEs that led to death, two of which were suspected to be related to the study treatment; one due to sepsis suspected to be related to avadomide, and the other due to progressive multifocal leukoencephalopathy suspected to be related to rituximab.

Of the 55 DLT‐evaluable patients, two experienced at least one toxicity meeting the DLT criteria. One patient with DLBCL experienced grade 4 neutropenia, which was suspected to be related to avadomide. One patient with FL experienced two occurrences of grade 3 diarrhea; the first was suspected to be related to avadomide, and the second was suspected to be related to both avadomide and rituximab.

### 3.4 Efficacy

**FIGURE 1:**
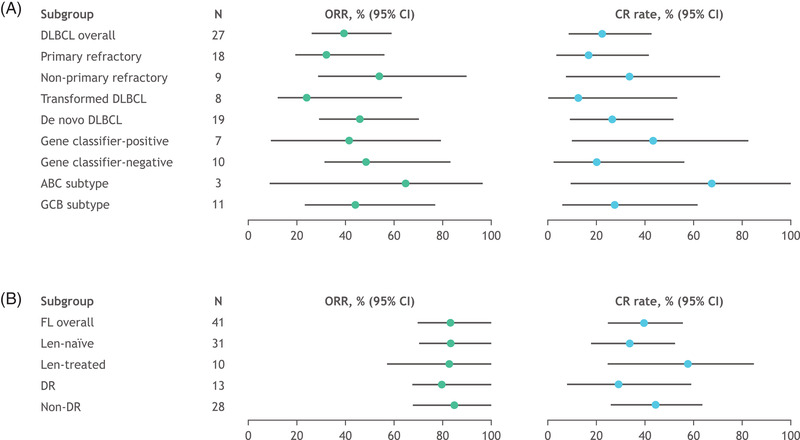
Efficacy across diffuse large B‐cell lymphoma (DLBCL) and follicular lymphoma (FL) subgroups. Forest plots presenting overall response rate (ORR) and complete response (CR) rate of (A) DLBCL and (B) FL subgroups. Data cutoff: January 10, 2020. Data presented are from the safety population. Abbreviations: ABC, activated B‐cell; CR, complete response; DLBCL, diffuse large B‐cell lymphoma; DR, double‐refractory; FL, follicular lymphoma; GCB, germinal center B‐cell; Len, lenalidomide; ORR, overall response rate

**FIGURE 2:**
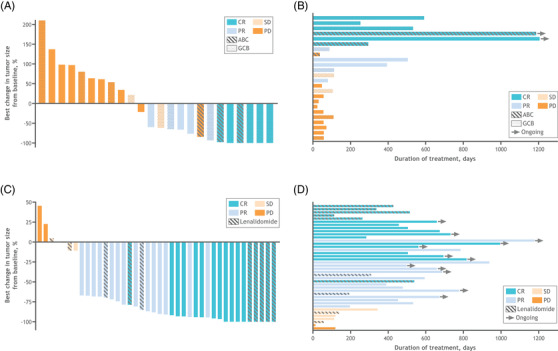
Best change in lesion size from baseline and duration of treatment. Best change in lesion size (A) and duration of treatment (B) by (A) best overall response and different cell of origin in patients with DLBCL, and best change in lesion size (C) and duration of treatment (D) by the best overall response and prior lenalidomide treatment status in patients with FL. Abbreviations: ABC, activated B‐cell; CR, complete response; DLBCL, diffuse large B‐cell lymphoma; FL, follicular lymphoma; GCB, germinal center B‐cell; Len, lenalidomide; PD, progressive disease; PR, partial response; SD, stable disease

**FIGURE 3:**
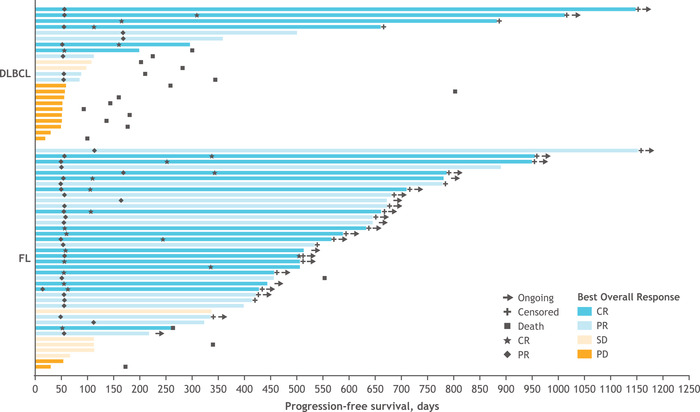
Progression‐free survival by the best overall response (safety population). Swim plot presenting duration of progression‐free survival and best overall response in patients with DLBCL and FL. Data cutoff: January 10, 2020. Data presented are from the safety population. Abbreviations: CR, complete response; DLBCL, diffuse large B‐cell lymphoma; FL, follicular lymphoma; PD, progressive disease; PR, partial response; SD, stable disease

Among the 27 patients with R/R DLBCL, the overall response rate (ORR) was 40.7%, with 6 patients (22.2%) achieving a CR (Figures 1A and 2A,B; Table S5). The median time to best overall response was 3.7 months (95% CI, 1.8–5.6). mDOR was 8.0 months (95% CI, 1.1–not evaluable [NE]), and median OS was 7.4 months. Median PFS was 1.9 months (95% CI, 1.7–3.7; Figure 3). As of January 10, 2020, the median duration of follow‐up for patients with R/R DLBCL was 6.9 months. In the overall FL cohort, the ORR was 80.5%, including 17 patients (41.5%) achieving a CR (Figures 1B and 2C,D; Table S6). The median time to best overall response was 1.9 months (95% CI, 1.8–3.5). mDOR was 27.6 months (95% CI, 16.7–NE), and median PFS was 22.1 months (95% CI, 15.0–NE; Figure 3). Median OS was not reached at the time of data cutoff. Progression of disease within 24 months occurred in 14 of 41 (34.1%) FL patients. As of January 10, 2020, the median duration of follow‐up for patients with R/R FL was 22.9 months.

Subgroup analysis conducted in patients with R/R DLBCL showed that the ORR was higher in non‐primary refractory DLBCL (55.6%) compared with primary refractory DLBCL (33.3%). Likewise, the response rate was higher in de novo DLBCL (47.4%) compared with transformed DLBCL (25.0%) (*p *< 0.05). There were no statistically significant differences in response rates based on gene classifier status or COO (Table S5). Median PFS was longer in de novo DLBCL (2.9 months [95% CI, 1.7–6.5]) compared with transformed DLBCL (1.8 months [95% CI, 0.6–16.4]). Among patients with R/R FL, ORR was similar in lenalidomide‐naïve and lenalidomide‐treated patients; 80.6% and 80.0%, respectively (Figure 1; Table S6). Median PFS was 29.2 months (95% CI, 16.6–NE) in lenalidomide‐naïve patients and 14.5 months (95% CI, 2.2–25.6) in lenalidomide‐treated patients. mDOR was 14.9 months (95% CI, 6.9–23.8) in lenalidomide‐treated patients and not reached in lenalidomide‐naïve patients.

### 3.5 Pharmacokinetics

Mean plasma concentration‐time profiles of avadomide are shown in Figure S2. Patients with DLBCL and FL had similar pharmacokinetic profiles on cycle 1 days 1 and 15. The mean avadomide concentration at 1.5 h reached 77.71 and 80.17 ng/ml for DLBCL and 77.59 and 74.53 ng/ml for FL, on days 1 and 15, respectively. Pharmacokinetic data showed no clinically meaningful impact of tumor type on avadomide drug exposure. Moderate to high between‐patient variability was noted and ranged from 42.2% to 168.2%.

## 4 DISCUSSION

The treatment of NHL continues to improve, with targeted agents, immunomodulatory imide drugs, and CAR T‐cell therapies resulting in significant improvements in survival in patients with R/R NHL [8, 25–32]. However, there is still a need for additional treatment options. The dose‐expansion part of this phase Ib study examined 3 mg avadomide given orally dosed 5/7 days per week in combination with 375 mg/m^2^ rituximab administered intravenously in 28‐day cycles or 3‐month intervals in a cohort of patients with R/R DLBCL or R/R FL [21].

The 5/7‐day dosing regimen was designed based on previous avadomide clinical studies, including the dose‐escalation portion of the current study, to mitigate neutropenia by releasing a reversible arrest in neutrophil maturation caused by depletion of Ikaros [20, 33]. Neutropenia was the most common grade 3/4 TEAE overall, seen as early as cycle 1, and was expected based on the mechanism of action of avadomide. These TEAEs were effectively managed with G‐CSF or GM‐CSF treatment or by dose interruptions or reductions. Grades 3 or 4 febrile neutropenia rates were low because neutropenia was well monitored and managed.

Multiple studies have shown that patients with ABC subtype DLBCL have significantly poorer outcomes with standard upfront rituximab‐containing chemoimmunotherapy compared with GCB disease; in the R/R setting, the prognostic impact of COO remains less clear [2]. Some recent therapeutic options are based on COO, which can be used independently of the IPI, to determine optimal treatment strategy in the R/R setting [2, 34–36]. A phase II/III study in patients with R/R DLBCL showed improved median PFS in response to lenalidomide monotherapy in the non‐GCB subtype [9]. In a phase II trial, the BTK inhibitor ibrutinib elicited higher ORRs in patients with the ABC subtype compared with the GCB subtype (37% vs. 5%, respectively) [35]. Results from the dose‐expansion phase suggest that responses to avadomide in combination with rituximab were COO‐independent, with ORRs of 66.7% and 45.5% in patients with ABC and GCB subtype DLBCL, respectively, compared with 40.7% in the overall DLBCL population. Although the small number of patients for whom COO data were available (*n*  = 14) limits interpretation of this result, it is supported by data from preclinical in vitro and in vivo models, in which avadomide demonstrated broad activity across DLBCL COO status [15]. In a previous study of avadomide in patients with R/R DLBCL, separation of patients into two groups based on immune cell composition in the tumor microenvironment using a gene expression classifier predicted clinical benefit from avadomide monotherapy, with prolonged PFS and higher ORR in patients with classifier‐positive disease compared with patients with classifier‐negative disease [24, 33]. Preliminary efficacy results from the current study found no significant difference in ORR based on gene expression classifier status; however, patients with classifier‐positive disease had a higher CR rate (42.9% vs. 20.0%) and showed a trend toward longer median PFS (3.2 months vs. 2.8 months) and mDOR (NR [95% CI, 4.7–NE] vs. 1.9 months [95% CI, 1.0–NE]) compared with patients with classifier‐negative disease (Table S5). It should be noted that the sample size for each subgroup may have been too small to demonstrate differences.

In patients with R/R DLBCL and FL, avadomide in combination with rituximab demonstrated promising antitumor activity, particularly in patients with R/R FL. Interestingly, ORRs were similar between lenalidomide‐naïve and lenalidomide‐treated patients with FL. These results highlight the promising clinical activity of the combination, independent of prior treatment with lenalidomide, and the potential of the high potency of avadomide to overcome resistance to lenalidomide. Patients with double‐refractory FL also appeared to have a similar ORR to the overall population. In terms of PFS, avadomide appeared to be promising for patients with R/R FL. In the current study, median PFS in patients with R/R FL was 22.1 months (95% CI, 15.0–NE) with a 12‐month PFS rate of 73.1% (95% CI, 56.7–84.1). Although this observation is with a small patient population, it can be considered potentially clinically relevant in the context of data from other agents, such as the 12‐month PFS rate of 77.5% (95% CI, 66.6–85.2) observed in the ZUMA‐5 trial of axi‐cel in patients with R/R indolent NHL [27]. Patients with R/R indolent lymphoma treated with copanlisib, a pan‐class I PI3K inhibitor, had a median PFS of 11.2 months (95% CI, 8.1–17.6); patients treated with another PI3K inhibitor, idelalisib, had a median PFS of 11.0 months (95% CI, 8.0–14.0) [3, 28]. Tazemetostat, an oral inhibitor of EZH2, demonstrated a median PFS of 13.8 months (95% CI, 10.7–22.0) for patients with mutant EZH2 and 11.1 months (95% CI, 3.7–14.6) in those with wildtype EZH2 [37].

Several recent trials have demonstrated that rational targeted, chemotherapy‐free combinations may be beneficial to patients with R/R FL and DLBCL [38, 39]. The Fc‐enhanced CD19 monoclonal antibody tafasitamab plus lenalidomide resulted in an ORR of 60% with a 43% CR rate in patients with R/R DLBCL ineligible for ASCT [38]. Real‐world data confirmed the superiority of the tafasitamab and lenalidomide combination (ORR of 67.1%) compared with lenalidomide monotherapy (ORR of 34.2%) in R/R DLBCL [40]; the significantly higher ORR, CR, and OS indicated potential synergistic effects of this immunotherapy combination [41]. Clinical activity was observed with ibrutinib plus rituximab plus lenalidomide in R/R DLBCL, with an ORR of 44% and an mDOR of 15.9 months; the ORR was higher in patients with non‐GCB DLBCL than in patients with GCB DLBCL [32]. In the phase II GALEN trial of obinutuzumab in combination with lenalidomide in R/R DLBCL, the ORR was 35% and the CR rate was 18% with a well‐tolerated safety profile [42]. Based on the findings from the phase III AUGMENT study, lenalidomide plus rituximab (R^2^) was recently approved in R/R FL. This combination demonstrated an ORR of 78%, with a CR rate of 34% in a patient population comprising R/R marginal zone lymphoma (17%) and R/R FL (83%) [39]. In the phase II GALEN trial of lenalidomide and obinutuzumab in R/R FL, the ORR was 79% with a CR rate of 38%, and the combination had a manageable safety profile [43]. The combination of obinutuzumab and zanubrutinib, a next‐generation BTK inhibitor, resulted in an ORR of 72% and a CR rate of 39% in patients with R/R FL [44]. These studies demonstrate the growing role of a chemotherapy‐free approach in R/R NHL and support the rationale for further evaluation, such as randomized trials that use correlative biomarkers to identify optimal therapeutic strategies and studies that explore potential novel synergistic combinations.

In conclusion, avadomide with rituximab was generally well tolerated. The safety profile of avadomide in combination with rituximab was consistent with the known safety profile of each respective study drug. No new safety signals were observed, and the rate of dose reductions was expected, supporting the 5/7‐day dosing regimen. Preliminary results show that this combination has promising antitumor activity in patients with R/R DLBCL and FL. Taken together, findings from this study support the potential of CELMoD agents in combination with anti‐CD20 monoclonal antibodies as a novel chemotherapy‐free treatment option for patients with R/R DLBCL and FL.

## FUNDING INFORMATION

Study funding was provided by Celgene, a Bristol Myers Squibb company.

## CONFLICT OF INTEREST

Loretta J. Nastoupil received honoraria from ADC Therapeutics, Bayer, Celgene, a Bristol Myers Squibb Company, Epizyme, Genentech, Gilead, Janssen, MorphoSys, Novartis, Pfizer, and TG Therapeutics; and received research funding from Bristol Myers Squibb/Celgene, Epizyme, Genentech, Janssen, Novartis, and TG Therapeutics. John Kuruvilla received honoraria from Amgen, Antengene, AstraZeneca, Celgene, a Bristol Myers Squibb company Gilead, Incyte, Janssen, Karyopharm, Merck, Novartis, Pfizer, Roche, Seattle Genetics, and TG Therapeutics; served as a consultant or advisor for AbbVie, Bristol Myers Squibb, Gilead, Karyopharm, Merck, Roche, and Seattle Genetics; and received research funding from AstraZeneca, Janssen, Roche, and Merck. Julio C. Chavez served as a consultant or advisor for AstraZeneca, Bayer, Celgene, a Bristol Myers Squibb Company, Genentech, Karyopharm, Morphosys, Novartis, Verastem, and Pfizer; received research funding from Merck; and served on the speaker's bureau for Genentech and AstraZeneca. Fontanet Bijou served as consultant or advisor for Bristol Myers Squibb and AbbVie. Armando Santoro served as consultant or advisor for Arqule, Bayer, Bristol Myers Squibb, Eisai, Gilead, Merck Sharp & Dohme, Pfizer, Sanofi, and Servier, and served on speaker's bureau for AbbVie, Amgen, Arqule, AstraZeneca, Bayer, Bristol Myers Squibb, Celgene, a Bristol Myers Squibb Company, Eisai, Gilead, Lilly, Merck Sharp & Dohme, Novartis, Pfizer, Roche, Sandoz, Servier, and Takeda. Ian W. Flinn served as consultant or advisor for AbbVie, AstraZeneca, BeiGene, Genentech, Gilead Sciences, Great Point Partners, Iksuda Therapeutics, Janssen, Juno Therapeutics, Kite Pharma, MorphoSys, Novartis, Nurix Therapeutics, Pharmacyclics, Roche, Seattle Genetics, Takeda, TG Therapeutics, Unum Therapeutics, Verastem, and Yingli Pharmaceuticals; and received research funding from AbbVie, Acerta Pharma, Agios, ArQule, AstraZeneca, BeiGene, Calithera Biosciences, Celgene, a Bristol Myers Squibb Company, Constellation Pharmaceuticals, Curis, Forma Therapeutics, Forty‐Seven, Genentech, Gilead Sciences, IGM Biosciences, Incyte, Infinity Pharmaceuticals, Janssen, Juno Therapeutics, Karyopharm Therapeutics, Kite Pharma, Loxo, Merck, MorphoSys, Novartis, Pfizer, Pharmacyclics, Portola Pharmaceuticals, Rhizen Pharmaceuticals, Roche, Seattle Genetics, Takeda, Teva, TG Therapeutics, Trillium Therapeutics, Triphase Research & Development Corp., Unum Therapeutics, and Verastem. Vaishalee P. Kenkre received research funding from AbbVie, Celgene, a Bristol Myers Squibb Company, MEI Pharma, and Novartis. P. Corradini received honoraria from AbbVie, ADC Therapeutics, Amgen, Celgene, a Bristol Myers Squibb Company, Daiichi Sankyo, Gilead, Incyte, Janssen, Kite, KyowaKirin, Novartis, Roche, Sanofi, and Takeda; served as consultant or advisor for AbbVie, ADC Therapeutics, Amgen, Celgene, a Bristol Myers Squibb Company, Daiichi Sankyo, Gilead, Incyte, Janssen, Kite, KyowaKirin, Novartis, Roche, Sanofi, and Takeda; and received travel funding from Novartis, Janssen, Celgene, a Bristol Myers Squibb Company, Bristol Myers Squibb, Takeda, Gilead, Amgen, and AbbVie. Iris Isufi served as a consultant or advisor for AstraZeneca, Bayer, Celgene, a Bristol Myers Squibb Company, Epizyme, and Kite. David J. Andorsky served as consultant or advisor for AbbVie and Bristol Myers Squibb and received research funding from AstraZeneca, Celgene, a Bristol Myers Squibb company, and Epizyme. Daniel Greenwald served as consultant or advisor for AstraZeneca and served on the speaker's bureau for Genentech and Jazz. R. Sangha received honoraria from AbbVie, AstraZeneca, Bristol Myers Squibb, Boehringer Ingelheim, Eli‐Lilly, Merck, Mylan, Pfizer, Roche/Genentech, Sanofi, Takeda, and Teva, and served as consultant or advisor for AbbVie, AstraZeneca, Bristol Myers Squibb, Boehringer Ingelheim, Eli‐Lilly, Merck, Roche/Genentech, Sanofi, Takeda, and Teva. Patrick Hagner, Nian Gong, Shailaja Uttamsingh, and Michael Pourdehnad are employed by and have equity ownership with Bristol Myers Squibb. Frank Shen, Yan Li, and Juergen Dobmeyer are employed by Bristol Myers Squibb. Vincent Ribrag received honoraria from AstraZeneca, Bristol Myers Squibb, Incyte, Merck Sharp & Dohme, Novartis, and Roche; served as a consultant or advisor for AstraZeneca, Bristol Myers Squibb, Merck Sharp & Dohme, Novartis, and Roche; received research funding from argenX, Astex, and GlaxoSmithKline; and received travel funding from AstraZeneca. No disclosures were reported by the other authors.

## AUTHOR CONTRIBUTIONS

**Loretta J. Nastoupil**: Conceptualization and design, data curation, formal analysis, investigation, methodology, writing original draft, project administration, writing review, and editing. **John Kuruvilla**: Data curation, formal analysis, investigation, methodology, writing original draft, project administration, writing review, and editing. **Julio C. Chavez**: Data curation, formal analysis, investigation, methodology, writing original draft, project administration, writing review, and editing. **Fontanet Bijou**: Data curation, formal analysis, investigation, methodology, writing original draft, project administration, writing review, and editing. **Thomas E. Witzig**: Data curation, formal analysis, investigation, methodology, writing original draft, project administration, writing review, and editing. **Armando Santoro**: Data curation, formal analysis, investigation, methodology, writing original draft, project administration, writing review, and editing. **Ian W. Flinn**: Data curation, formal analysis, investigation, methodology, writing original draft, project administration, writing review, and editing. **Carola Boccomini**: Data curation, formal analysis, investigation, methodology, writing original draft, project administration, writing review, and editing. **Vaishalee P. Kenkre**: Data curation, formal analysis, investigation, methodology, writing original draft, project administration, writing review, and editing. **Paolo Corradini**: Data curation, formal analysis, investigation, methodology, writing original draft, project administration, writing review, and editing. **Iris Isufi**: Data curation, formal analysis, investigation, methodology, writing original draft, project administration, writing review, and editing. **David J. Andorsky**: Data curation, formal analysis, investigation, methodology, writing original draft, project administration, writing review, and editing. **Leonard M. Klein**: Data curation, formal analysis, investigation, methodology, writing original draft, project administration, writing review, and editing. **Daniel R. Greenwald**: Data curation, formal analysis, investigation, methodology, writing original draft, project administration, writing review, and editing. **Randeep Sangha**: Data curation, formal analysis, investigation, methodology, writing original draft, project administration, writing review, and editing. **Frank Shen**: Data curation, formal analysis, investigation, methodology, writing original draft, project administration, writing review, and editing. **Patrick Hagner**: Data curation, formal analysis, investigation, methodology, writing original draft, project administration, writing review, and editing. **Yan Li**: Data curation, formal analysis, investigation, methodology, writing original draft, project administration, writing review, and editing. **Juergen Dobmeyer**: Data curation, formal analysis, investigation, methodology, writing original draft, project administration, writing review, and editing. **Nian Gong**: Data curation, formal analysis, investigation, methodology, writing original draft, project administration, writing review, and editing. **Shailaja Uttamsingh**: Conceptualization and design, data curation, formal analysis, investigation, methodology, writing original draft, project administration, writing review, and editing. **Michael Pourdehnad**: Conceptualization and design, study supervision, data curation, formal analysis, investigation, methodology, writing original draft, project administration, writing review, and editing. **Vincent Ribrag**: Conceptualization and design, data curation, formal analysis, investigation, methodology, writing original draft, project administration, writing review, and editing.

## Supporting information

SUPPORTING INFORMATION

## References

[1] Armitage JO , Gascoyne RD , Lunning MA , Cavalli F . Non‐Hodgkin lymphoma. Lancet 2017;390:298–310. 28153383 10.1016/S0140-6736(16)32407-2

[2] Liu Y , Barta SK . Diffuse large B‐cell lymphoma: 2019 update on diagnosis, risk stratification, and treatment. Am J Hematol. 2019;94:604–16. 30859597 10.1002/ajh.25460

[3] Salles G , Schuster SJ , de Vos S , Wagner‐Johnston ND , Viardot A , Blum KA , et al. Efficacy and safety of idelalisib in patients with relapsed, rituximab‐ and alkylating agent‐refractory follicular lymphoma: a subgroup analysis of a phase 2 study. Haematologica 2017;102:e156–e9. 27979923 10.3324/haematol.2016.151738 PMC5395130

[4] Salles GA . Clinical features, prognosis and treatment of follicular lymphoma. Hematol Am Soc Hematol Educ Program. 2007:216–25. 10.1182/asheducation-2007.1.216 18024633

[5] Thandra KC , Barsouk A , Saginala K , Padala SA , Barsouk A , Rawla P . Epidemiology of non‐Hodgkin's lymphoma. Med Sci. 2021;9:5. 10.3390/medsci9010005 PMC7930980 33573146

[6] Alizadeh AA , Eisen MB , Davis RE , Ma C , Lossos IS , Rosenwald A , et al. Distinct types of diffuse large B‐cell lymphoma identified by gene expression profiling. Nature 2000;403:503–11. 10676951 10.1038/35000501

[7] Chao MP . Treatment challenges in the management of relapsed or refractory non‐Hodgkin's lymphoma ‐ novel and emerging therapies. Cancer Manag Res. 2013;5:251–69. 24049458 10.2147/CMAR.S34273 PMC3775637

[8] National Comprehensive Cancer Network . NCCN clinical practice guidelines in oncology (NCCN Guidelines) B‐cell lymphomas, version 5. 2021. https://www.nccn.org/professionals/physician_gls/pdf/b‐cell.pdf. Accessed November 17, 2021.

[9] Czuczman MS , Trneny M , Davies A , Rule S , Linton KM , Wagner‐Johnston N , et al. A phase 2/3 multicenter, randomized, open‐label study to compare the efficacy and safety of lenalidomide versus investigator's choice in patients with relapsed or refractory diffuse large B‐cell lymphoma. Clin Cancer Res. 2017;23:4127–37. 28381416 10.1158/1078-0432.CCR-16-2818 PMC8171498

[10] Gisselbrecht C , Glass B , Mounier N , Singh Gill D , Linch DC , Trneny M , et al. Salvage regimens with autologous transplantation for relapsed large B‐cell lymphoma in the rituximab era. J Clin Oncol. 2010;28:4184–90. 20660832 10.1200/JCO.2010.28.1618 PMC3664033

[11] Horn H , Ziepert M , Becher C , Barth TF , Bernd HW , Feller AC , et al. MYC status in concert with BCL2 and BCL6 expression predicts outcome in diffuse large B‐cell lymphoma. Blood 2013;121:2253–63. 23335369 10.1182/blood-2012-06-435842

[12] Casulo C , Byrtek M , Dawson KL , Zhou X , Farber CM , Flowers CR , et al. Early relapse of follicular lymphoma after rituximab plus cyclophosphamide, doxorubicin, vincristine, and prednisone defines patients at high risk for death: An analysis from the National LymphoCare Study. J Clin Oncol. 2015;33:2516–22. 26124482 10.1200/JCO.2014.59.7534 PMC4879714

[13] Wang L , Li LR , Young KH . New agents and regimens for diffuse large B cell lymphoma. J Hematol Oncol. 2020;13:175. 33317571 10.1186/s13045-020-01011-z PMC7734862

[14] Hanel W , Epperla N . Evolving therapeutic landscape in follicular lymphoma: a look at emerging and investigational therapies. J Hematol Oncol. 2021;14:104. 34193230 10.1186/s13045-021-01113-2 PMC8247091

[15] Hagner PR , Man HW , Fontanillo C , Wang M , Couto S , Breider M , et al. CC‐122, a pleiotropic pathway modifier, mimics an interferon response and has antitumor activity in DLBCL. Blood 2015;126:779–89. 26002965 10.1182/blood-2015-02-628669 PMC4528065

[16] Cubillos‐Zapata C , Cordoba R , Avendano‐Ortiz J , Arribas‐Jimenez C , Hernandez‐Jimenez E , Toledano V , et al. CC‐122 immunomodulatory effects in refractory patients with diffuse large B‐cell lymphoma. Oncoimmunology 2016;5:e1231290. 28255524 10.1080/2162402X.2016.1231290 PMC5325046

[17] Gandhi AK , Vincent R , Carpio C , Stoppa A‐M , Gharibo MM , Damian S , et al. CC‐122 expands activated and memory CD4 and CD8 T cells in vivo and induces T cell activation ex vivo in subjects with relapsed or refractory diffuse large B cell lymphoma and multiple myeloma. Blood 2015;126:2704.26337492

[18] Zhang LH , Kosek J , Wang M , Heise C , Schafer PH , Chopra R . Lenalidomide efficacy in activated B‐cell‐like subtype diffuse large B‐cell lymphoma is dependent upon IRF4 and cereblon expression. Br J Haematol. 2013;160:487–502. 23252516 10.1111/bjh.12172

[19] Michot JM , Bouabdallah R , Vitolo U , Doorduijn JK , Salles G , Chiappella A , et al. Avadomide plus obinutuzumab in patients with relapsed or refractory B‐cell non‐Hodgkin lymphoma (CC‐122‐NHL‐001): a multicentre, dose escalation and expansion phase 1 study. Lancet Haematol. 2020;7:e649–e59. 32758434 10.1016/S2352-3026(20)30208-8

[20] Rasco DW , Papadopoulos KP , Pourdehnad M , Gandhi AK , Hagner PR , Li Y , et al. A first‐in‐human study of novel cereblon modulator avadomide (CC‐122) in advanced malignancies. Clin Cancer Res. 2019;25:90–8. 30201761 10.1158/1078-0432.CCR-18-1203

[21] Ribrag V , Chavez JC , Boccomini C , Kaplan J , Chandler JC , Santoro A , et al. Phase Ib study of combinations of Avadomide (CC‐122), CC‐223, CC‐292, and Rituximab in patients with relapsed/refractory diffuse large B‐cell lymphoma. ejhaem. 2022;3:139–53. 10.1002/jha2.375 PMC9176062 35846221

[22] Hatake K , Chou T , Doi T , Terui Y , Kato H , Hirose T , et al. Phase I, multicenter, dose‐escalation study of avadomide in adult Japanese patients with advanced malignancies. Cancer Sci. 2021;112:331–8. 33075165 10.1111/cas.14704 PMC7780008

[23] Cheson BD , Pfistner B , Juweid ME , Gascoyne RD , Specht L , Horning SJ , et al. Revised response criteria for malignant lymphoma. J Clin Oncol. 2007;25:579–86. 17242396 10.1200/JCO.2006.09.2403

[24] Risueno A , Hagner PR , Towfic F , Fontanillo C , Djebbari A , Parker JS , et al. Leveraging gene expression subgroups to classify DLBCL patients and select for clinical benefit from a novel agent. Blood 2020;135:1008–18. 31977005 10.1182/blood.2019002414 PMC7099333

[25] Chavez JC , Bachmeier C , Kharfan‐Dabaja MA . CAR T‐cell therapy for B‐cell lymphomas: clinical trial results of available products. Ther Adv Hematol. 2019;10:2040620719841581. 31019670 10.1177/2040620719841581 PMC6466472

[26] Chiappella A , Vitolo U . Lenalidomide in diffuse large B‐cell lymphomas. Adv Hematol. 2012;2012:498342. 22792112 10.1155/2012/498342 PMC3390034

[27] Jacobson C , Chavez JC , Sehgal AR , William BM , Munoz J , Salles G , et al. Primary analysis of Zuma‐5: A phase 2 study of axicabtagene ciloleucel (axi‐cel) in patients with relapsed/refractory (R/R) indolent non‐Hodgkin lymphoma (iNHL). Blood 2020;136:40–1.

[28] Dreyling M , Santoro A , Mollica L , Leppa S , Follows GA , Lenz G , et al. Phosphatidylinositol 3‐kinase inhibition by copanlisib in relapsed or refractory indolent lymphoma. J Clin Oncol. 2017;35:3898–905. 28976790 10.1200/JCO.2017.75.4648

[29] Schuster SJ , Bishop MR , Tam CS , Waller EK , Borchmann P , McGuirk JP , et al. Tisagenlecleucel in adult relapsed or refractory diffuse large B‐cell lymphoma. N Engl J Med. 2019;380:45–56. 30501490 10.1056/NEJMoa1804980

[30] Locke FL , Ghobadi A , Jacobson CA , Miklos DB , Lekakis LJ , Oluwole OO , et al. Long‐term safety and activity of axicabtagene ciloleucel in refractory large B‐cell lymphoma (ZUMA‐1): a single‐arm, multicentre, phase 1–2 trial. Lancet Oncol. 2019;20:31–42. 30518502 10.1016/S1470-2045(18)30864-7 PMC6733402

[31] Abramson JS , Palomba ML , Gordon LI , Lunning MA , Wang M , Arnason J , et al. Lisocabtagene maraleucel for patients with relapsed or refractory large B‐cell lymphomas (TRANSCEND NHL 001): a multicentre seamless design study. Lancet 2020;396:839–52. 32888407 10.1016/S0140-6736(20)31366-0

[32] Goy A , Ramchandren R , Ghosh N , Munoz J , Morgan DS , Dang NH , et al. Ibrutinib plus lenalidomide and rituximab has promising activity in relapsed/refractory non–germinal center B‐cell–like DLBCL. Blood. 2019;134:1024–36. 31331917 10.1182/blood.2018891598 PMC6764267

[33] Carpio C , Bouabdallah R , Ysebaert L , Sancho JM , Salles G , Cordoba R , et al. Avadomide monotherapy in relapsed/refractory DLBCL: safety, efficacy, and a predictive gene classifier. Blood 2020;135:996–1007. 31977002 10.1182/blood.2019002395 PMC7099331

[34] Xu‐Monette ZY , Zhang H , Zhu F , Tzankov A , Bhagat G , Visco C , et al. A refined cell‐of‐origin classifier with targeted NGS and artificial intelligence shows robust predictive value in DLBCL. Blood Adv. 2020;4:3391–404. 32722783 10.1182/bloodadvances.2020001949 PMC7391158

[35] Wilson WH , Young RM , Schmitz R , Yang Y , Pittaluga S , Wright G , et al. Targeting B cell receptor signaling with ibrutinib in diffuse large B cell lymphoma. Nat Med. 2015;21:922–6. 26193343 10.1038/nm.3884 PMC8372245

[36] Scott DW , Mottok A , Ennishi D , Wright GW , Farinha P , Ben‐Neriah S , et al. Prognostic significance of diffuse large B‐cell lymphoma cell of origin determined by digital gene expression in formalin‐fixed paraffin‐embedded tissue biopsies. J Clin Oncol. 2015;33:2848–56. 26240231 10.1200/JCO.2014.60.2383 PMC4554747

[37] Morschhauser F , Tilly H , Chaidos A , McKay P , Phillips T , Assouline S , et al. Tazemetostat for patients with relapsed or refractory follicular lymphoma: an open‐label, single‐arm, multicentre, phase 2 trial. Lancet Oncol. 2020;21:1433–42. 33035457 10.1016/S1470-2045(20)30441-1 PMC8427481

[38] Salles G , Duell J , González Barca E , Tournilhac O , Jurczak W , Liberati AM , et al. Tafasitamab plus lenalidomide in relapsed or refractory diffuse large B‐cell lymphoma (L‐MIND): a multicentre, prospective, single‐arm, phase 2 study. Lancet Oncol. 2020;21:978–88. 32511983 10.1016/S1470-2045(20)30225-4

[39] Leonard JP , Trneny M , Izutsu K , Fowler NH , Hong X , Zhu J , et al. AUGMENT: a phase III study of lenalidomide plus rituximab versus placebo plus rituximab in relapsed or refractory indolent lymphoma. J Clin Oncol. 2019;37:1188–99. 30897038 10.1200/JCO.19.00010 PMC7035866

[40] Rodgers T , Luigi Zinzani P , Marino D , Frezzato M , Maria Barbui A , Castellino C , et al. ABCL‐135: RE‐MIND: a comparison of tafasitamab (MOR208) + lenalidomide (L‐MIND) versus lenalidomide monotherapy (real‐world data) in transplant‐ineligible patients with relapsed/refractory diffuse large B‐cell lymphoma. Clin Lymphoma Myeloma Leuk. 2020;20:S265–S6.

[41] Nowakowski GS , Rodgers TD , Marino D , Frezzato M , Barbui AM , Castellino C , et al. RE‐MIND study: a propensity score‐based 1:1 matched comparison of tafasitamab + lenalidomide (L‐MIND) versus lenalidomide monotherapy (real‐world data) in transplant‐ineligible patients with relapsed/refractory (R/R) diffuse large B‐cell lymphoma (DLBCL). J Clin Oncol. 2020;38:Abstract 8020.

[42] Houot R , Cartron G , Bijou F , de Guibert S , Salles GA , Fruchart C , et al. Obinutuzumab plus Lenalidomide (GALEN) for the treatment of relapse/refractory aggressive lymphoma: a phase II LYSA study. Leukemia 2019;33:776–80. 30291335 10.1038/s41375-018-0282-y

[43] Morschhauser F , Le Gouill S , Feugier P , Bailly S , Nicolas‐Virelizier E , Bijou F , et al. Obinutuzumab combined with lenalidomide for relapsed or refractory follicular B‐cell lymphoma (GALEN): a multicentre, single‐arm, phase 2 study. Lancet Haematol. 2019;6:e429–e37. 31296423 10.1016/S2352-3026(19)30089-4

[44] Tam CS , Quach H , Nicol A , Badoux X , Rose H , Prince HM , et al. Zanubrutinib (BGB‐3111) plus obinutuzumab in patients with chronic lymphocytic leukemia and follicular lymphoma. Blood Adv. 2020;4:4802–11. 33022066 10.1182/bloodadvances.2020002183 PMC7556127

