# Supplementary material for: Phase Ib study of avadomide (CC‐122) in combination with rituximab in patients with relapsed/refractory diffuse large B‐cell lymphoma and follicular lymphoma
Source: EJHaem. 2022 Feb 14;3(2):394–405. doi: 10.1002/jha2.394 (PMC9175947; doi:10.1002/jha2.394)
Supplement: Supplementary file 1 — SUPPORTING INFORMATION [file JHA2-3-394-s001.docx]

## SUPPLEMENTARY MATERIALS

Supplementary materials include supplementary methods, tables (patient disposition, treatment duration, modification, and exposure [safety population], treatment-emergent adverse events in patients with DLBCL and FL, treatment-emergent serious adverse events in patients with DLBCL and FL, efficacy by DLBCL subgroup, and efficacy by FL group), and figures (study design and plasma concentration–time profiles of avadomide), in pdf format.

## Supplementary Methods

### Patients

Eligible patients were men and women who were at least 18 years old and had histologically or cytologically confirmed disease. The DLBCL cohort included patients with chemorefractory DLBCL, including transformed low-grade lymphoma, which is defined as SD or PD as best response to the last chemotherapy-containing regimen, or disease progression or recurrence ≤12 months after prior ASCT. Patients must have received adequate prior therapy such as anti-CD20 monoclonal antibody unless tumor was determined to be CD20-negative and an anthracycline-containing chemotherapy regimen; at least 1 prior line of salvage therapy unless ineligible for autologous transplant; and prior chemotherapy for FL in patients with transformed FL. Patients must have been ineligible for ASCT at the time of enrollment.

The FL cohort included patients with CD20-positive R/R FL. The lenalidomide-naïve cohort received at least 1 prior standard systemic treatment regimen including systemic chemo-, immune-, or chemo-immunotherapy and at least 1 prior line of salvage therapy with no prior exposure to lenalidomide, or double-refractory with no prior exposure to lenalidomide. Double refractory patients were refractory to both rituximab and an alkylating agent. The lenalidomide-exposed cohort received at least 2 cycles of lenalidomide-containing regimen, either as a single agent or in combination, and experienced early relapse or early progression after lenalidomide treatment or were disease refractory to lenalidomide. Lenalidomide or lenalidomide-containing regimen did not need to be the immediate prior regimen received for eligibility in the study.

Additional inclusion criteria were measurable disease >1.5 cm in the long axis or >1.0 cm in both long and short axes, an Eastern Cooperative Oncology Group performance status score of 0 or 1, adequate hematologic function (absolute neutrophil count ≥1.5 x 10^9^/L without growth factor support for 7 days [14 days if patients received pegfilgrastim], hemoglobin ≥8 g/dL, platelets ≥100 x 10^9^/L without transfusion for 7 days), hepatic function (serum bilirubin ≤1.5 x upper limit of normal [ULN], alanine aminotransferase and aspartate aminotransferase ≤2.5 x ULN or 5.0 x ULN if liver tumor was present), and renal function (estimated serum creatinine 24-hour clearance ≥50 mL/min), and potassium levels within normal limits or correctable with supplements. Exclusion criteria include symptomatic central nervous system involvement; known acute or chronic pancreatitis; persistent diabetes or malabsorption despite medical management; peripheral neuropathy grade ≥2 per National Cancer Institute Common Terminology Criteria for AEs (NCI CTCAE); impaired cardiac function or clinically significant cardiac diseases; diabetes; prior ASCT ≤3 months before first dose; prior allogeneic SCT with standard or reduced intensity conditioning; prior systemic anticancer treatments ≤5 half-lives or 4 weeks before the start of study drug, whichever was shorter; major surgery ≤2 weeks before starting study drugs; pregnant or breast-feeding women; known HIV, HBV, or HCV infection; treatment-related myelodysplastic syndrome or a history of concurrent secondary cancers requiring active, ongoing treatment; or any other significant medical condition, laboratory abnormality, or psychiatric illness that would cause unacceptably high risk or that would prevent the subject from complying with the study.

### Dose-limiting toxicities (DLTs)

Dose-limiting toxicities were defined as follows:

- Any grade 4 nonhematologic toxicity of any duration during cycle 1 that was suspected to be related to study treatment
- Grade 3 bilirubin elevation, whether symptomatic or asymptomatic, and other hepatic laboratory abnormalities, including the following:
  - Elevated bilirubin at screening due to Gilbert’s syndrome or hemolysis if total bilirubin increased to 2 times the highest study-recorded pretreatment value or met grade 3 criteria
  - Transaminase elevation ≥3 times the ULN
  - Total bilirubin more than 2 times the highest study-recorded pretreatment value in the absence of other medical reasons or concomitant medication
- Clinically relevant nonhematologic toxicity that was suspected to be related to study drugs and that occurred during cycle 1 and was grade 3 with the following exceptions:
  - Grade 3 acneiform, pustular, or maculopapular rash that resolved to grade ≤2 within 7 days of dose interruption and did not recur at the same level upon resumption of study treatment at the same dose level (with medical management)
  - Grade 3 diarrhea or vomiting lasting <3 days (with medical management)
  - Grade 3 fatigue or oral mucositis/stomatitis that resolved to grade ≤2 within 7 days of dose interruption and did not recur at the same level upon resumption of study treatment at the same dose level (with medical management)
  - Tumor lysis syndrome that did not progress to grade 4 and resolved within 7 days with medical management
  - A nonhematologic grade 3 clinical laboratory adverse event that was asymptomatic and rapidly reversible (ie, returned to baseline or grade ≤1 within 7 days)
- Hyperglycemia meeting the following criteria:
  - Grade 2 fasting hyperglycemia lasting >14 days
  - Grade ≥3 hyperglycemia lasting >4 days
  - Grade 4 hyperglycemia lasting ≥12 hours despite optimal medical treatment
  - Hyperglycemia associated with diabetic ketoacidosis or nonketonic hyperosmolar coma regardless of glucose level
- Hematological toxicities as follows:
  - Any febrile neutropenia
  - Grade 4 neutropenia lasting >7 days
  - Grade 4 thrombocytopenia lasting >24 hours
  - Grade 3 or 4 thrombocytopenia with clinically significant bleeding
- Any AE suspected to be treatment-related and necessitating dose reduction during cycle 1

**Table S1.** Patient disposition.

|  | **DLBCL** | **FL overall** | **Overall** |
| --- | --- | --- | --- |
|  | **(*N* = 27)** | **(*N* = 41)** | **(*N* = 68)** |
| Patients still on treatment, *n* (%) | 2 (7.4) | 12 (29.3) | 14 (20.6) |
| Discontinued, *n* (%) | 25 (92.6) | 29 (70.7) | 54 (79.4) |
| Reasons for discontinuation, *n* (%) |  |  |  |
| Progressive disease | 18 (66.7) | 11 (26.8) | 29 (42.6) |
| Adverse event^a^ | 6 (22.2) | 10 (24.4) | 16 (23.5) |
| Physician decision | 0 | 6 (14.6) | 6 (8.8) |
| Death | 0 | 1 (2.4) | 1 (1.5) |
| Other | 1 (3.7) | 1 (2.4) | 2 (2.9) |

Data cutoff: January 10, 2020.
*DLBCL* diffuse large B-cell lymphoma, *FL* follicular lymphoma.

^a^ Includes 2 patients with adverse events not considered to be treatment emergent. Treatment-emergent adverse events are defined as any adverse event occurring or worsening on or after the first treatment of the study drug, and within 28 days after the last dose of the study drug received.

**Table S2.** Treatment duration, modification, and exposure (safety population).

|  | **DLBCL** | **Len-naïve** | **Len-treated** | **FL overall** | **Overall** |
| --- | --- | --- | --- | --- | --- |
|  | **(*N* = 27)** | **(*N* = 31)** | **(*N* = 10)** | **(*N* = 41)** | **(*N* = 68)** |
| Median overall treatment duration (range), d | 80 (8-1206) | 533 (14-1178) | 286.5 (57-539) | 498 (14-1178) | 323.5 (8-1206) |
| **Avadomide** |  |  |  |  |  |
| Median duration (range),^a^ d | 62.0  (7-1199) | 531.0  (14-1171) | 244.5  (39-514) | 488.0  (14-1171) | 274.5  (7-1199) |
| Median no. of cycles (range) | 2.0 (0-42) | 17.0 (0-41) | 7.0 (0-18) | 16.0 (0-41) | 9.0 (0-42) |
| Median relative dose intensity (range), mg/d | 1.00 (0.7-1.0) | 0.99 (0.7-1.0) | 0.98 (0.5-1.0) | 0.98 (0.5-1.0) | 0.99 (0.5-1.0) |
| ≥ 1 dose reduction, *n* (%) | 3 (11.1) | 7 (22.6) | 5 (50.0) | 12 (29.3) | 15 (22.1) |
| Adverse event | 2 (7.4) | 7 (22.6) | 5 (50.0) | 12 (29.3) | 14 (20.6) |
| Other | 1 (3.7) | 0 | 0 | 0 | 1 (1.5) |
| Missing | 0 | 0 | 1 (10.0) | 1 (2.4) | 1 (1.5) |
| ≥ 1 dose interruption, *n* (%) | 24 (88.9) | 28 (90.3) | 9 (90.0) | 37 (90.2) | 61 (89.7) |
| Adverse event | 17 (63.0) | 24 (77.4) | 7 (70.0) | 31 (75.6) | 48 (70.6) |
| Other | 16 (59.3) | 23 (74.2) | 8 (80.0) | 31 (75.6) | 47 (69.1) |
| **Rituximab** |  |  |  |  |  |
| Median duration (range),^b^ d | 77.0 (21-581) | 301.0 (21-609) | 203.0 (49-273) | 273.0 (21-609) | 245.0 (21-609) |
| Median no. of cycles (range) | 3.0 (1-21) | 11.0 (1-22) | 7.5 (2-10) | 10.0 (1-22) | 9.0 (1-22) |
| Median relative dose intensity (range), mg/d | 0.99 (0.9-1.1) | 0.98 (0.9-1.0) | 0.99 (0.9-1.0) | 0.99 (0.9-1.0) | 0.99 (0.9-1.1) |
| ≥ 1 dose interruption, *n* (%) | 13 (48.1) | 23 (74.2) | 6 (60.0) | 29 (70.7) | 42 (61.8) |
| Adverse event | 11 (40.7) | 15 (48.4) | 5 (50.0) | 20 (48.8) | 31 (45.6) |
| Other | 4 (14.8) | 19 (61.3) | 5 (50.0) | 24 (58.5) | 28 (41.2) |

Data cutoff: January 10, 2020.
^a^ Defined as [(Last nonzero dose date of avadomide in cycle) – (First nonzero dose date of avadomide in cycle) + 2 + 1].
^b^ Defined as [21 + 28 × (Number of subsequent cycles where rituximab was administered after Cycle 1)].
*d* day *DLBCL* diffuse large B-cell lymphoma *FL* follicular lymphoma *Len* lenalidomide.

**Table S3.** Treatment-emergent adverse events in patients with DLBCL and FL.

|  | **Any-grade** | **Grade 3/4** |
| --- | --- | --- |
|  | **(*N* = 68)** | **(*N* = 68)** |
| **Hematologic,** *n* (%) |  |  |
| Neutropenia | 44 (64.7) | 39 (57.4) |
| Anemia | 15 (22.1) | 5 (7.4) |
| Febrile neutropenia | 7 (10.3) | 6 (8.8) |
| Lymphopenia | 5 (7.4) | 4 (5.9) |
| **Gastrointestinal** |  |  |
| Nausea | 18 (26.5) | 0 |
| Diarrhea | 15 (22.1) | 2 (2.9) |
| Vomiting | 14 (20.6) | 1 (1.5) |
| Constipation | 14 (20.6) | 0 |
| Abdominal pain | 11 (16.2) | 3 (4.4) |
| Anal ulcer | 1 (1.5) | 1 (1.5) |
| Obstructive pancreatitis | 1 (1.5) | 1 (1.5) |
| **Other** |  |  |
| Cough | 25 (36.8) | 0 |
| Fatigue | 24 (35.3) | 6 (8.8) |
| Pyrexia | 17 (25.0) | 0 |
| Back pain | 17 (25.0) | 0 |
| Overdose | 16 (23.5) | 0 |
| Edema peripheral | 14 (20.6) | 0 |
| Muscle spasms | 14 (20.6) | 0 |
| Pruritus | 14 (20.6) | 0 |
| Rash | 14 (20.6) | 1 (1.5) |
| Lipase increased | 7 (10.3) | 6 (8.8) |

Data cutoff: January 10, 2020.
Any-grade TEAEs reported in ≥20% of patients (DLBCL or FL) or grade 3/4 TEAEs reported in ≥5% of patients.
*FL* follicular lymphoma, *TEAE* treatment-emergent adverse event.

**Table S4.** Treatment-emergent serious adverse events occurring in patients with DLBCL and FL.

|  | **DLBCL overall** | **FL overall** | **Overall** |
| --- | --- | --- | --- |
|  | **(*N* = 27)** | **(*N* = 41)** | **(*N* = 68)** |
| **≥1 Any-grade SAE, *n* (%)** | 12 (44.4) | 20 (48.8) | 32 (47.1) |
| Febrile neutropenia | 1 (3.7) | 4 (9.8) | 5 (7.4) |
| Pneumonia | 1 (3.7) | 3 (7.3) | 4 (5.9) |
| Acute kidney injury | 0 | 2 (4.9) | 2 (2.9) |
| General physical health deterioration | 2 (7.4) | 0 | 2 (2.9) |
| Influenza | 1 (3.7) | 1 (2.4) | 2 (2.9) |
| Progressive multifocal leukoencephalopathy | 2 (7.4) | 0 | 2 (2.9) |
| Pyrexia | 0 | 2 (4.9) | 2 (2.9) |
| **≥1 Any-grade avadomide-related SAE, *n* (%)** | 1 (3.7) | 10 (24.4) | 11 (16.2) |
| Febrile neutropenia | 0 | 3 (7.3) | 3 (4.4) |
| Pneumonia | 0 | 2 (4.9) | 2 (2.9) |
| Anemia | 0 | 1 (2.4) | 1 (1.5) |
| Progressive multifocal leukoencephalopathy | 1 (3.7) | 0 | 1 (1.5) |
| Rectal abscess | 0 | 1 (2.4) | 1 (1.5) |
| Sepsis | 0 | 1 (2.4) | 1 (1.5) |
| Ejection fraction decreased | 0 | 1 (2.4) | 1 (1.5) |
| Arthritis | 0 | 1 (2.4) | 1 (1.5) |
| Acute kidney injury | 0 | 1 (2.4) | 1 (1.5) |
| Bronchopneumopathy | 0 | 1 (2.4) | 1 (1.5) |
| Pneumonitis | 0 | 1 (2.4) | 1 (1.5) |
| Deep vein thrombosis | 0 | 1 (2.4) | 1 (1.5) |

Data cutoff: January 10, 2020.
Serious adverse events reported in ≥2 patients overall and avadomide-related serious adverse events reported in ≥1 patient.
*DLBCL* diffuse large B-cell lymphoma, *FL* follicular lymphoma, *SAE* serious adverse event.

**Table S5.** Efficacy by DLBCL subgroup.

| **Efficacy outcome** | **DLBCL  Overall** | **Primary refractory DLBCL** | **Non-primary refractory DLBCL** | ***P* value** | **Transformed DLBCL** | **De novo  DLBCL** | ***P* value** | **Gene classifier-positive** | **Gene classifier-negative** | ***P* value** | **ABC subtype** | **GCB subtype** | ***P* value** |
| --- | --- | --- | --- | --- | --- | --- | --- | --- | --- | --- | --- | --- | --- |
|  | **(*N* = 27)** | **(*N* = 18)** | **(*N* = 9)** |  | **(*N* = 8)** | **(*N* = 19)** |  | **(*N* = 7)** | **(*N* = 10)** |  | **(*N* = 3)** | **(*N* = 11)** |  |
| ORR, % (95% CI)^a^ | 40.7 (22.4-61.2) | 33.3 (13.3-59.0) | 55.6 (21.2-86.3) | 0.411 | 25.0 (3.2-65.1) | 47.4 (24.4-71.1) | 0.019 | 42.9 (9.9-81.6) | 50.0 (18.7-81.3) | 1.00 | 66.7 (9.4-99.2) | 45.5 (16.7-76.6) | 1.00 |
| CR, %  (95% CI) | 22.2 (8.6-42.3) | 16.7 (3.6-41.4) | 33.3 (7.5-70.1) |  | 12.5 (0.3-52.7) | 26.3 (9.1-51.2) |  | 42.9 (9.9-81.6) | 20.0 (2.5-55.6) |  | 66.7 (9.4-99.2) | 27.3 (6.0-61.0) |  |
| PR, *n* (%) | 5 (18.5) | 3 (16.7) | 2 (22.2) |  | 1 (12.5) | 4 (21.1) |  | 0 | 3 (30.0) |  | 0 | 2 (18.2) |  |
| Median time to best overall response, mo (95% CI) | 3.7 (1.8-5.6) | 2.8 (1.8-10.2) | 5.3 (1.8-5.6) |  | 3.7 (1.9-5.6) | 3.7 (1.8-5.6) |  | 3.7 (1.8-5.5) | 1.8 (1.8-5.3) |  | 3.6 (1.9-5.3) | 1.8 (1.8-5.5) |  |
| SD, *n* (%) | 2 (7.4) | 1 (5.6) | 1 (11.1) |  | 0 | 2 (10.5) |  | 1 (14.3) | 1 (10.0) |  | 0 | 2 (18.2) |  |
| PD, *n* (%) | 12 (44.4) | 9 (50.0) | 3 (33.3) |  | 6 (75.0) | 6 (31.6) |  | 2 (28.6) | 4 (10.0) |  | 1 (33.3) | 3 (27.3) |  |
| Missing, *n* (%) | 2 (7.4) | 2 (11.1) | 0 |  | 0 | 2 (10.5) |  | 1 (14.3) | 0 |  | 0 | 1 (9.1) |  |
| mPFS (95% CI), mo | 1.9 (1.7–3.7) | 1.8 (1.6–2.9) | 6.5 (0.7–16.4) | 0.408 | 1.8 (0.6–16.4) | 2.9 (1.7–6.5) | 0.017 | 3.2 (1.0–NE) | 2.8 (0.7–3.7) | 0.458 | 9.7 (0.7–NE) | 3.2 (1.6–6.5) | 0.523 |
| mDOR (95% CI), mo | 8.0 (1.1–NE) | NR (1.0–NE) | 8.0 (4.7–NE) | 0.764 | NR (10.9–NE) | 6.3 (1.0–NE) | 0.908 | NR (4.7-NE) | 1.9 (1.0–NE) | 0.204 | NR (8.0–NE) | 4.7 (1.1–NE) | 0.588 |
| mOS (95% CI), mo | 7.4  (5.3–26.4) | 7.4  (4.5–11.3) | 9.9 (3.3–NE) | 0.246 | 6.0  (1.0–NE) | 8.0 (4.8–11.3) | 0.013 | 9.6 (1.6–NE) | 6.9 (3.3–NE) | 0.475 | NR (3.3–NE) | 7.2 (1.6–9.9) | 0.299 |

Data cutoff: January 10, 2020.
Data presented are from the safety population.
*ABC* activated B-cell, *CR* complete response, *DLBCL* diffuse large B-cell lymphoma, *DR* double-refractory, *FL* follicular lymphoma, *GCB* germinal center B-cell, *Len* lenalidomide, *mDOR* median duration of response, *mOS* median overall survival, *mPFS* median progression-free survival, *NE* not evaluable, *NR* not reached, *ORR* overall response rate, *PD* progressive disease, *PR* partial response, *SD* stable disease.
^a^ Response as determined by the investigator based on International Working Group Criteria for malignant lymphoma.

**Table S6.** Efficacy by FL group.

| **Efficacy outcome** | **FL  overall** | **Len-naïve** | **Len-treated** | **DR** | **Non-DR** | ***P* value^a^** |
| --- | --- | --- | --- | --- | --- | --- |
|  | **(*N* = 41)** | **(*N* = 31)** | **(*N* = 10)** | **(*N* = 13)** | **(*N* = 28)** |  |
| ORR, % (95% CI) | 80.5 (65.1-91.2) | 80.6 (62.5-92.5) | 80.0 (44.4-97.5) | 76.9  (46.2-95.0) | 82.1  (63.1-93.9) | 0.693 |
| CR, % (95% CI) | 41.5 (26.3-57.9) | 35.5 (19.2-54.6) | 60.0 (26.2-87.8) | 30.8 (9.1-61.4) | 46.4 (27.5-66.1) |  |
| PR, *n* (%) | 16 (39.0) | 14 (45.2) | 2 (20.0) | 6 (46.2) | 10 (35.7) |  |
| Median time to best overall response, mo (95% CI) | 1.9 (1.8-3.5) | 1.9 (1.8-3.7) | 1.9 (1.7-3.6) | 2.7 (1.6-3.7) | 1.9 (1.8-2.1) |  |
| SD, *n* (%) | 5 (12.2) | 3 (9.7) | 2 (20.0) | 2 (15.4) | 3 (10.7) |  |
| PD, *n* (%) | 3 (7.3) | 3 (9.7) | 0 | 1 (7.7) | 2 (7.1) |  |
| Missing, n (%) | 0 | 0 | 0 | 0 | 0 |  |
| mPFS (95% CI), mo | 22.1 (15.0–NE) | 29.2 (16.6–NE) | 14.5 (2.2–25.6) | NR (3.7–NE) | 22.1 (13.1–NE) | 0.667 |
| mDOR (95% CI), mo | 27.6 (16.7–NE) | NR (19.3–NE) | 14.9 (6.9–23.8) | NR (5.6–NE) | 23.8 (14.9-NE) | 0.349 |
| mOS (95% CI), mo | NR | NR | NR  (8.7–NE) | NR (18.2–NE) | NR | 0.059 |

Data cutoff: January 10, 2020.
*CR* complete response, *DLBCL* diffuse large B-cell lymphoma, *DR* double-refractory, *Len* lenalidomide, *FL* follicular lymphoma, *mDOR* median duration of response, *mOS* median overall survival, *mPFS* median progression-free survival, *NE* not evaluable, *NR* not reached, *ORR* overall response rate, *PD* progressive disease, *PR* partial response, *SD* stable disease.
^a^ Comparison between DR and non-DR patients.

**Fig. S1 Study design.** CC-122-DLBCL-001 is a multicenter, open-label, phase Ib study. The dose-expansion portion of this study, highlighted in the orange box, examined avadomide in combination with rituximab in patients with relapsed/refractory DLBCL or FL.


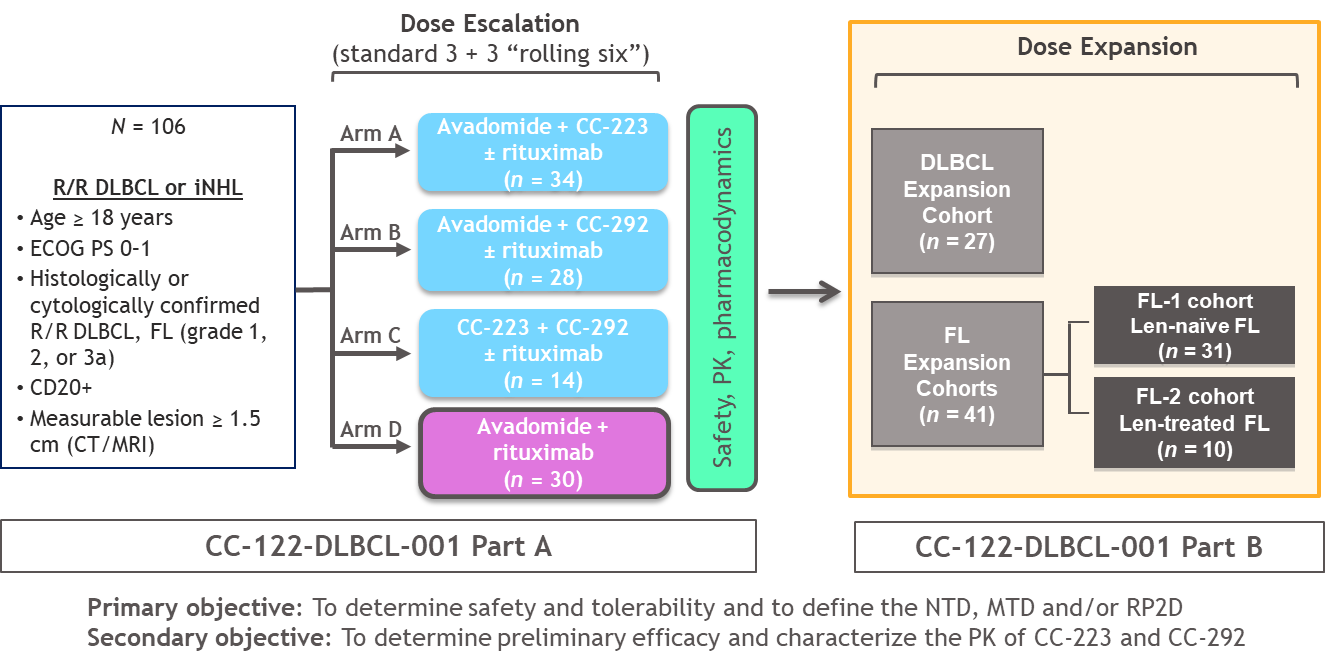


CT/MRI computed tomography/magnetic resonance imaging, DLBCL diffuse large B-cell lymphoma, ECOG PS Eastern Cooperative Oncology Group performance status, FL follicular lymphoma, Len lenalidomide, MTD maximum-tolerated dose, NTD non-tolerated dose, PK pharmacokinetics, RP2D recommended phase 2 dose, R/R relapsed/refractory.

**Fig. S2 Plasma concentration–time profiles of avadomide.** Mean avadomide plasma concentration– time profiles on a linear scale for **A** cycle 1 day 1 and **B** cycle 1 day 15.

**A.**

**B.**
